# Commentary: Psychology's Questionable Research Fundamentals (QRFs): key problems in quantitative psychology and psychological measurement beyond Questionable Research Practices (QRPs)

**DOI:** 10.3389/fpsyg.2026.1752466

**Published:** 2026-02-24

**Authors:** Mario S. Staller, Swen Koerner

**Affiliations:** 1 Department of Police, University for Police and Public Administration North Rhine-Westphalia, Cologne, Germany; 2 Department of Training Pedagogy and Martial Research, German Sports University Cologne, Cologne, Germany

**Keywords:** autopoietic psychic systems, psychological measurement, reflexive psychology, systems theory, theory crisis

Uher et al. (2025) provide a timely contribution to the current debate about psychology's crises. Rather than treating replication failures, validity problems, and generalisability issues as local defects to be repaired by incremental reforms, the authors argue that these crises originate in the discipline's research fundamentals—its largely implicit ontological, epistemological, and methodological commitments. In doing so, they provide the grounds for “rethinking psychology as a science” (p. 2) and for advancing philosophy-of-science work that can integrate psychology's fragmented empirical landscape.

In this commentary, we pursue three aims. First, we highlight where the paper's diagnoses align with a system-theoretical account of the psyche as an autopoietic, self-referential system (Luhmann, 1995a; Staller and Koerner, 2025). Second, we suggest how a system-theoretical perspective can unify several strands of critique that the authors treat in parallel. Third, we sketch an outlook: what follows if we not only repair psychology's fundamentals but also shift its central object from “psychological entities” to psychology's own ways of observing and constructing them? Our contribution aims to raise psychology's reflexive potential by treating QRFs not as isolated methodological deficits, but as a coherent pattern that recurrently arises when operatively closed, meaning-processing psychic systems are approached through measurement formats presupposing external accessibility and stable conceptualisations of psychological properties. What systems theory adds beyond generic calls for conceptual clarity is a mechanism-level account of operational closure and self-referential meaning production: psychological “properties” are not simply found but become stabilized through the distinctions an observing practice imposes. This reframes QRFs as recurrent consequences of how measurement couples to meaning-processing systems, rather than as isolated technical flaws of instruments or statistics. A concrete implication for quantitative work is interpretive: scores and effects should be treated as outcomes of instrument–context–respondent coupling, and claims of cross-situational or cross-person comparability should be stated explicitly as conditional on demonstrated invariance and stable interpretive frames.

From our perspective, four strands of Uher et al.'s (2025) analysis are particularly consequential, and each can be re-specified in system-theoretical terms by making explicit (a) the implicit assumption diagnosed, (b) the reinterpretation, and (c) a cautious implication for measurement and inference.

First, the critique of scientism, quantificationism, and statisticism diagnoses the assumption that numerical output secures scientificity. From a system-theoretical perspective, quantification is an observational reduction that selects and stabilizes distinctions in a meaning medium. This becomes visible when high model fit, small *p*-values, or “objective” scale scores are treated as evidence that the underlying psychological entity is well-captured, whilst the meaning-distinction that makes the variable measurable remains implicit. Accordingly, statistical sophistication may be treated as insufficient evidence for ontic structure unless the distinctions enabling quantification are explicitly justified.

Second, the discussion of measurement theories and psychometric practice targets the assumption—typical of strong measurement interpretations—that psychological attributes behave like stable quantities. Systems theory instead suggests that, under operational closure, instruments do not “read off” inner magnitudes but stabilize communicable formats of meaning. This is evident when Likert-type ratings (e.g., “Anxiety” 1–5) are interpreted as if they were interval-scaled quantities, or when latent-variable scores are treated as person-properties independent of context, despite well-known influences of response styles, testing situations, and item framing in the obtained numbers. Thus, scores should be interpreted as instrument- and context-bound constructions, and comparability claims should be stated as conditional (here, “metrological standards” refer to strong/fundamental measurement requirements such as evidence for quantity structure and meaningful unit concatenation, not to pragmatic statistical modeling *per se*).

Third, the analysis of language, items, and constructs diagnoses the assumption that item responses transparently indicate underlying entities. System-theoretically, responses are context-sensitive selections shaped by wording and self-reference. This is apparent when minor wording changes (e.g., “often” vs. “frequently”) shift endorsement, when item meanings differ across groups or contexts, or when socially desirable responding systematically reconfigures response patterns—showing that item semantics and context are constitutive parts of what is being “measured.” Hence, item semantics and context should be treated as constitutive parts of the measurement model rather than as mere noise, and constructs should not be reified from indicator patterns alone.

Fourth, the treatment of ergodicity and sample-to-individual inference diagnoses the assumption that between-person structure generalizes to within-person dynamics. From a system-theoretical angle, non-ergodicity is expected when meaning-processing reorganizes across time and contexts. This matters whenever associations observed between individuals (e.g., higher average stress correlation with poorer average sleep) are used to infer within-person causal dynamics, even though the within-person pattern may differ or even reverse across situations and time. Therefore, individual-level claims should be restricted when derived from aggregate statistics, and within-person designs/analyses should be preferred when individuals are the target.

From a system-theoretical perspective on psychic systems, these four strands do not appear as isolated problems but as expressions of a deeper structural mismatch. Systems theory treats the psyche not as a container of inner objects that can be inspected and measured from the outside, but as an autopoietic system whose basic operation is the ongoing production of experiential distinctions (Luhmann, 1995a). The system is operationally closed (Luhmann, 1995b): it does not receive fully formed inputs from an external world, but continuously selects, formats, and interprets differences in its own medium of meaning. Many of the difficulties highlighted by Uher et al. (2025) are thus exactly what one would expect when a discipline attempts to treat such a system as if it were a measurable object in an externally accessible domain.

This structural mismatch becomes most visible once the critique is translated into metrological terms. In other words, the very standards that define measurement in the strict sense presuppose forms of stability and homogeneity that meaning-processing psychic systems systematically undermine. Measurement in the metrological sense presupposes stable, concatenable quantities defined as homogeneous qualities. The experiential operations of psychic systems, however, do not exhibit these properties. They are historically, biographically, and situationally saturated selections of meaning that cannot be decomposed into additive units without losing precisely what makes them psychic. The observation that psychometrics struggles to connect numerals to meaningful structures is therefore not an accidental weakness, but a systematic consequence of an observational translation problem: psychometric numbers are outputs of an observing practice that must translate meaning-based operations into comparable units, whilst the relevant “structures” are themselves interpretation- and context-dependent. The same holds for the contextuality of psychological phenomena and the limitations of laboratory experimentation that the authors discuss: The gap between experimental findings and lived experience is not simply due to poor ecological validity, but rooted in the attempt to isolate variables in ways that are structurally at odds with the emergent, context-dependent organization of experiential processes.

From this angle, the QRFs identified by the authors can be read as manifestations of one underlying pattern: psychology is attempting to handle psychic systems with conceptual and methodological tools that presuppose external accessibility. These points directly toward the outlook that Uher et al. (2025) call for when they argue that rethinking psychology as a science requires advancing its philosophy-of-science theories. One possible direction is to shift psychology's primary object (Staller and Koerner, 2025). Instead of treating “the psyche” as a pre-given entity that is insufficiently understood or imperfectly measured, psychology could treat as its main object how it brings “psyche” into form: through distinctions, concepts, measurement instruments, designs, and inferential practices (see, e.g., Hutmacher and Franz, 2025, for the vagueness of psychological concepts).

Our system-theoretical reading of the authors' studies would therefore not oppose their critique, but radicalize its central insight. It would ask psychology to include its own observing in what it observes: to take seriously that the discipline does not simply discover pre-existing psychological entities, but actively participates in their formation and stabilization. Rather than seeking a final, unified foundation for psychological science, it would explore how different observational logics—experimental, psychometric, qualitative, historical—produce different “psychologies” and how their limits and paradoxes can be rendered visible without disabling further research. In this sense, Uher et al. (2025)'s article can be read as an important step toward a more reflexive psychology. A system-theoretical perspective on autopoietic psychic systems and on psychology as an observing practice offers one possible path in that space. On a practical level, the suggested system-theoretical view would turn “measurement failure” into a diagnosable observation problem: it directs attention to how instruments stabilize meanings (and thus entities) through specific distinctions, rather than treating mismatches as mere technical noise. This kind of shift is not a rhetorical add-on: it changes what counts as an explanatory target (the observing practice) and which methodological demands become appropriate (e.g., conditional comparability, context-sensitive modeling, and design choices aligned with meaning-processing).
